# VEGF inhibitor‐induced vascular dysfunction involves redox‐sensitive PARP activation and SIRT1 disruption

**DOI:** 10.1113/EP093180

**Published:** 2026-06-17

**Authors:** Karla B. Neves, Rheure Alves‐Lopes, Augusto C. Montezano, Rhian M. Touyz

**Affiliations:** ^1^ Strathclyde Institute of Pharmacy & Biomedical Sciences University of Strathclyde Glasgow UK; ^2^ Institute of Cardiovascular and Medical Sciences University of Glasgow Glasgow UK; ^3^ School of Medicine, Medical Sciences and Nutrition University of Aberdeen Aberdeen UK; ^4^ Research Institute of the McGill University Health Centre Montreal Quebec Canada; ^5^ Department of Medicine and Department of Family Medicine McGill University Montreal Quebec Canada

**Keywords:** endothelial dysfunction, oxidative stress, PARP activation, sirtuin 1, vascular inflammation, VEGFR inhibition

## Abstract

Vascular endothelial growth factor receptor (VEGFR) inhibitors are effective antiangiogenic agents used in cancer therapy. However, they are associated with cardiovascular disease, including hypertension and vascular dysfunction. The molecular mechanisms underlying these cardiovascular toxicities are unclear, but oxidative stress might be important. Here we investigated the potential role of redox‐sensitive poly(ADP‐ribose) polymerase (PARP) and sirtuin 1 (SIRT1) in VEGFR inhibitor‐induced vascular injury. Molecular studies were performed in axitinib (VEGFR inhibitor)‐treated human aortic endothelial cells, and vascular studies were undertaken in isolated intact vessels from mice. Axitinib increased reactive oxygen species production and PARP activation. This was prevented by tiron (antioxidant) and olaparib (PARP inhibitor). Phosphorylation of endothelial nitric oxide synthase at Thr^495^ (inhibitory site) was increased and activity of SIRT1 was reduced following axitinib treatment. This was accompanied by increased p53 acetylation; these effects were mitigated by olaparib or the SIRT1 activator SRT1720. VEGFR inhibition increased expression and secretion of pro‐inflammatory markers (MCP‐1 and interleukin‐6) and adhesion molecules (VCAM‐1 and ICAM‐1) and promoted THP‐1 monocyte adhesion to human aortic endothelial cells, effects that were attenuated by PARP inhibition or SIRT1 activation. In isolated arteries, axitinib enhanced contractile responses to U46619 and endothelin‐1 while impairing ACh‐induced relaxation. Co‐treatment with olaparib or SRT1720 restored vascular responses and endothelial nitric oxide synthase phosphorylation. In conclusion, inhibition of VEGFR signalling induces oxidative stress and PARP activation, leading to SIRT1 downregulation, endothelial dysfunction and vascular inflammation. Targeting PARP activation or enhancing SIRT1 activity might represent promising strategies to mitigate VEGF inhibitor‐induced vascular complications.

## Abstract (highlights)

- **What is the central question of this study?**
  Vascular endothelial growth factor receptor (VEGFR) inhibitors are associated with cardiovascular toxicity, including vascular dysfunction, but the underlying mechanisms remain unclear. A role for redox‐sensitive PARP activation and disruption of SIRT1 signalling in this context has not previously been reported.
- **What is the main finding and its importance?**
  We show that inhibition of VEGFR induces oxidative stress and activates PARP, which reduces SIRT1 activity, leading to vascular inflammation and endothelial dysfunction. We identify the PARP–SIRT1 axis as a molecular mechanism underlying VEGFR inhibitor‐induced vascular injury and suggest that it might be a putative therapeutic target to mitigate unwanted vascular effects of VEGFR inhibitor therapy.

## 1 INTRODUCTION

Angiogenesis, the formation of new blood vessels from pre‐existing vasculature, is a fundamental process in both physiological and pathological contexts. From embryonic development and wound healing to tumour growth and metastasis, the formation of new blood vessels plays a pivotal role (Saman et al., 2020). Vascular endothelial growth factor (VEGF) is a key regulator of angiogenesis, orchestrating endothelial cell proliferation, migration, survival and vascular permeability (Lorenc et al., 2024). By binding its tyrosine kinase receptor on endothelial cells, VEGF triggers a cascade of intracellular signalling events that promote angiogenesis. Given its crucial role in driving angiogenesis, VEGF and its receptors (VEGFR) have emerged as prime targets for anti‐cancer therapies (Al‐Ostoot et al., 2021; Lorenc et al., 2024).

VEGF signalling inhibitors (VEGFi), including monoclonal antibodies, such as bevacizumab, and small molecule tyrosine kinase inhibitors (TKIs; e.g., axitinib, sunitinib and sorafenib), have gained widespread clinical use in the treatment of various malignancies (Escalante & Zalpour, 2011; Santorsola et al., 2024). Through their antiangiogenic actions, these agents effectively inhibit tumour growth and metastasis, offering significant clinical benefits for cancer patients (Wang et al., 2024). However, the clinical use of VEGFi is often hampered by the development of cardiovascular toxicities, including hypertension, thromboembolic events, heart failure, cardiomyopathies and arterial dysfunction (Alameddine et al., 2015; Escalante & Zalpour, 2011; le Noble et al., 2023; Mihalcea et al., 2023). These adverse effects not only diminish the quality of life of patients but also pose limitations on treatment duration (Faruque et al., 2014).

Molecular mechanisms underlying VEGFi‐induced cardiovascular toxicities remain incompletely understood. Recent studies suggest that VEGFi disrupts the balance of redox signalling in vascular tissues, leading to increased production of reactive oxygen species (ROS) and subsequent oxidative stress (Neves et al., 2018). This oxidative stress can activate various downstream pathways, including poly(ADP‐ribose) polymerase‐1 (PARP‐1), a DNA repair enzyme that, upon activation, consumes significant amounts of cellular NAD^+^ (Kauppinen et al., 2013; Martin‐Guerrero et al., 2020). The depletion of NAD^+^ might subsequently impair activity of sirtuin 1 (SIRT1), a NAD^+^‐dependent deacetylase known to play a crucial role in maintaining endothelial function and vascular tone (Sundaresan et al., 2011). Considering the intricate interplay between VEGFR inhibition, oxidative stress, PARP activation and SIRT1 signalling, we hypothesise that inhibition of VEGF signalling promotes oxidative stress and consequent PARP activation, leading to vascular dysfunction through disruption of SIRT1 signalling.

## 2 MATERIALS AND METHODS

### 2.1 Ethical approval

All experimental protocols on mice were performed in accordance with the United Kingdom Animals Scientific Procedures Act 1986 and ARRIVE Guidelines and approved by the University of Glasgow Animal Welfare and Ethics Review Board (approval no. 70/9021). Mice were housed in individual cages in a room with controlled humidity and temperature (22°C–24°C) and in light–dark cycles of 12 h–12 h, with ad libitum access to normal rodent chow and water. For ex vivo studies, mice were euthanized by Forane (isoflurane) anaesthesia overdose (4%–5%) via inhalation and exsanguinated via abdominal vein puncture.

### 2.2 Cell culture

For cell‐based studies, human aortic endothelial cells (HAECs; Promocell^®^, Germany; C‐12271) were used. HAECs were cultured in endothelial cell growth medium (Promocell^®^) supplemented with penicillin/streptomycin (50 µg⁄mL) and endothelial cell growth medium supplement (10 mL; Promocell^®^). For functional studies, confluent cells were made quiescent for 2 h in low‐serum medium containing 0.5% fetal bovine serum before stimulation. Only low‐passage cells (passages 4–8) were studied. Cells were exposed to axitinib (1 µmol/L), a VEGFR tyrosine kinase inhibitor that inhibits VEGFR signalling (Neves et al., 2023; SelleckChem S1005), in the absence or presence of pharmacological inhibitors [olaparib, 1 µmol/L (Alves‐Lopes et al., 2020; Glentham Life Sciences GP0126); tiron (ROS scavenger), 10 µmol/L or SIRT1 activator, SRT1720, 2 µmol/L (Milne et al., 2007; SelleckChem S1129)] for different time periods prior to stimulation with axitinib.

### 2.3 Isolated intact arteries

C57BL/6 mice were bred within the animal facility at the University of Glasgow. All experiments were performed in male and female mice randomly selected at 3 months of age. Mouse mesenteric resistance arteries (first and second order; ∼300–350 µm) were isolated from male C57BL/6 mice (10–12 weeks old). Mesenteric arteries were selected for study owing to their significance in peripheral resistance and their role in blood pressure regulation. All experiments were performed in randomly selected male and female mice.

Briefly, perivascular tissue was removed, arteries were cut into 2 mm ring segments and mounted on isometric wire myographs (Danish Myo Technology, Aarhus, Denmark) filled with 5 mL of physiological saline solution (in mmol/L: 130 NaCl, 14.9 NaHCO_3_, 4.7 KCl, 1.18KH_2_PO_4_, 1.17 MgSO_4_.7H_2_O, 5.5 glucose, 1.56 CaCl_2_.2H_2_O and 0.026 EDTA) and continuously gassed with a mixture of 95% O_2_ and 5% CO_2_ while being maintained at a constant temperature of 37°C. Following 30 min of equilibration, the contractile responses of arterial segments were assessed by the addition of KCl (62.5 mmol/L). The integrity of the endothelium was verified by relaxation induced by ACh (1 µmol/L) in arteries precontracted with a thromboxane A2 agonist (U46619) (0.1 µmol/L).

### 2.4 Myographic assessment of vascular functional

Arterial segments were mounted on a wire myograph (Danish Myo Technology, Aarhus, Denmark) filled with 5 mL of physiological solution and continuously gassed with a mixture of 95% O_2_ and 5% CO_2_ at 37°C. The relationship between resting wall tension and internal circumference was determined, and the internal circumference, L100, corresponding to a transmural pressure of 100 mmHg for a relaxed vessel in situ, was calculated. The vessels were set to the internal circumference, L1, given by: L1 = 0.9 × L100. The effective internal lumen diameter was determined as L1 = L1/π and was between 200 and 300 µm. After 30 min of stabilization, the contractile ability of the preparations was assessed by adding KCl solution (120 mM) to the organ baths. To check vascular contractility, a concentration–response curve was performed with U46619 (thromboxane A2 analogue), endothelin‐1 (ET‐1), ACh and sodium nitroprusside (SNP). When used, olaparib and SRT1720 were added 30 min prior to incubations with axitinib.

### 2.5 Measurement of ROS

NADPH‐mediated ROS generation in HAECs was measured by enhanced lucigenin chemiluminescence, with lucigenin as the electron acceptor and NADPH as the substrate (Griendling et al., 2016). Stimulated HAECs were washed with ice‐cold PBS and homogenized in lysis buffer (20 mmol/L of KH_2_PO_4_, 1 mmol/L of EGTA, 1 µg/mL of aprotinin, 1 µg/mL of leupeptin, 1 µg/mL of pepstatin and 1 mmol/L of phenylmethylsulphonyl fluoride). Fifty microlitres of the sample was added to a suspension containing 175 µL of assay buffer (50 mmol/L of KH_2_PO_4_, 1 mmol/L of EGTA and 150 mmol/L of sucrose, pH 7.4) and lucigenin (5 µmol/L). Luminescence was measured for 30 cycles of 18 s each by a luminometer (Orion II Microplate Luminometer, Berthold, Germany). Basal readings were obtained prior to the addition of NADPH (100 µmol/L) to the assay. The reaction was started by the addition of the substrate. Basal and buffer blank values were subtracted from the NADPH‐derived luminescence. ROS production was expressed as relative luminescence units (RLU) per microgram of protein.

### 2.6 Real‐time PCR

Total RNA was isolated from HAECs using the TRIzol^®^ reagent (Life Technologies) according to the manufacturer's instructions and diluted in nuclease‐free H_2_O (Ambion/Life Technologies, Paisley, UK). Complementary DNA was generated from total RNA using High‐Capacity cDNA Reverse Transcription Kits (Applied Biosystems, Warrington, UK). Real‐time PCR was performed with the Applied Biosystems 7900HT Fast Real‐Time PCR system, using Power SYBR Green Master Mix (Applied Biosystems) and specific human primers.

### 2.7 Immunoblotting

Total HAEC protein was extracted using 50 mmol/L Tris–HCl (pH 7.4) lysis buffer containing 1% Nonidet P‐40, 0.5% sodium deoxycholate, 150 mmol/L NaCl, 1 mmol/L EDTA, 0.1% SDS, 2 mmol/L sodium orthovanadate (Na_3_VO_4_), 1 mM phenylmethylsulphonyl fluoride, 1 µg/mL pepstatin A, 1 µg/mL leupeptin and 1 µg/mL aprotinin. Total protein extract was sonicated and cleared by centrifugation at 8,000 g for 10 min, and the pellet was discarded. The protein concentration was determined using the Thermo Scientific™ Pierce™ Bicinchoninic Acid (BCA) protein assay kit. Proteins from homogenates (30 µg) were separated by electrophoresis on a polyacrylamide gel and transferred onto a nitrocellulose membrane. Non‐specific binding sites were blocked with 3% bovine serum albumin in Tris‐buffered saline solution with 0.1 % Tween for 1 h at room temperature. Membranes were then incubated with specific antibodies overnight at 4°C. Antibodies were as follows: anti‐eNOS (Thr^495^) antibody (Cell Signaling 9574S, 1:1000 TBST–BSA 3%), anti‐eNOS (Ser^1177^) antibody (Cell Signaling 9571S, 1:1000 TBST–BSA 3%), anti‐acetyl‐p53 (Cell Signaling 2525S, 1:1000 TBST–BSA 3%) and anti‐β‐actin antibody (Sigma, A1978, 10:10 000 TBST–BSA 3%). After incubation (1 h) with respective secondary fluorescence‐coupled antibodies (LICOR^®^), signals were visualized by an infrared laser scanner (Odyssey Clx, LICOR^®^). Protein expression levels were normalized to loading controls from respective membranes.

### 2.8 SIRT1 activity

SIRT1 activity was quantified in stimulated HAECs using the Human SIRT1 ELISA Kit (Abcam, Cambridge, UK, catalogue no. ab171573). Undiluted samples were processed according to the manufacturer's instructions. Along with biological samples, the microplate contained positive controls (serial dilutions of the SIRT1 standard, serving to make the standard curve) and negative controls (sample dilution buffer). Based on standard curves generated for the assay, the SIRT1 concentration in the analysed samples was calculated from absorbance units, measured using a microplate spectrophotometer at a wavelength of 450 nm. Samples were assayed in duplicate, and the values were averaged.

### 2.9 PARP activity

PARP activity was assessed based on the detection of biotinylated poly (ADP‐ribose) deposited by PARP‐1 onto immobilized histones (R&D Systems^®^ 4677‐096‐K). Forty micrograms of of protein from cell lysates was loaded into a 96‐well plate coated with histones and biotinylated poly(ADP‐ribose), allowed to incubate for 1 h, treated with streptavidin‐horseradish peroxidase, and read at 450 nm in a spectrophotometer.

### 2.10 Monocyte adhesion assay

HAECs were cultured in 24‐well plates and stimulated with axitinib for 24 h, in the presence or absence of olaparib or SRT1720. Monocytes derived from human Tohoku Hospital Pediatrics‐1 (THP‐1) cells were obtained from American Type Culture Collection (Manassas, VA, USA). THP‐1 cell suspensions were cultured in cellular growth medium (RPMI with penicillin/streptomycin and 10% fetal bovine serum). Monocytes were suspended in saline solution supplemented with 0.1% bovine serum albumin containing 10 mmol/L of the CellTrace™ CFSE (Life Technologies^®^) and incubated for 20 min at 37°C. Stimulated HAECs were washed two times in PBS. Labelled monocytes (1 × 10^6^ cells per well) were added for 40 min at 37°C in 5% CO_2_, for eventual adhesion. After incubation, non‐adherent monocytes were gently removed by washing with PBS. Counting of adherent cells was performed by visualization in a fluorescence microscope. The number of monocytes was determined by the average number of adhered cells in three fields captured from each well.

### 2.11 Measurement of IL‐6 and MCP‐1 levels

Interleukin‐6 (IL‐6) and monocyte chemoattractant protein‐1 (MCP‐1) levels were assessed in the culture medium of HAECs using an ELISA protocol. The ELISA procedure involved coating an ELISA plate with specific capture antibodies targeting IL‐6 (R&D Systems DY206) and MCP‐1 (R&D Systems DY279), followed by incubation with diluted HAEC samples and standards. After washing steps, a substrate solution was added, and the reaction was terminated. The optical density of each well was measured at 450 nm, enabling the determination of IL‐6 and MCP‐1 concentrations by comparing the readings against a standard curve. The experiments included cells stimulated with axitinib in the presence or absence of olaparib and/or SRT1720.

### 2.12 Statistical analysis

For vascular functional studies, data were analysed by determining the EC_50_ and maximal response (*E*_max_) values from experimental data fitted to a four‐parameter logistic function against the null hypothesis. The *p*D_2_ (defined as the negative logarithm of the EC_50_ values) and *E*_max_ were compared by two‐way ANOVA with Bonferroni *post hoc* test, as appropriate. For other experiments, statistical comparisons between groups were performed using one‐way ANOVA with Dunnett's *post hoc* test. A value of *P* ≤ 0.05 was considered statistically significant. Data analysis was conducted using GraphPad Prism^®^ v.10.0 (GraphPad Software Inc., San Diego, CA, USA). Results are expressed as the mean ± SD. As indicated in the figure legends, each data point/number in the figures represents a different sample.

## 3 RESULTS

### 3.1 VEGFR inhibition increases oxidative stress and PARP activity in human aortic endothelial cells

**FIGURE 1:**
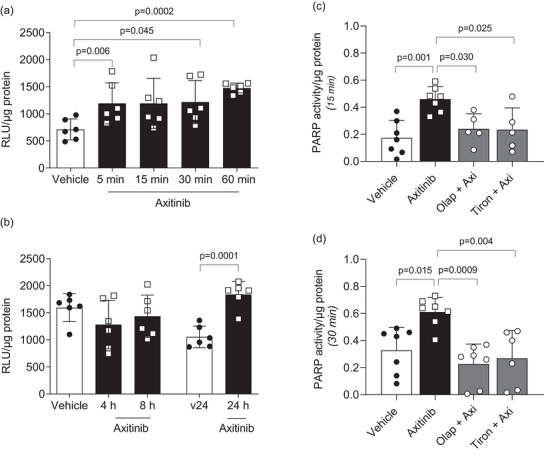
Axitinib, a VEGFR inhibitor, increases ROS production and PARP activity in human aortic endothelial cells. (a, b) Human aortic endothelial cells were treated with vehicle or axitinib for the indicated times, and ROS production was assessed by lucigenin‐enhanced chemiluminescence (*n =* 6). (c, d) PARP activity was measured by ELISA at 15 (*n =* 7) and 30 min (*n =* 7) after treatment. Where indicated, cells were pretreated with olaparib (Olap) or tiron for 30 min before axitinib exposure (15 min, *n =* 5; 30 min, *n =* 7 and *n =* 6, respectively). Results are expressed as the mean ± SD (one‐way ANOVA with *post hoc* test; Student's *t* test for 24 h group). Olap: olaparib; Axi: axitinib; RLU: relative light units; PARP: Poly (ADP‐ribose) polymerase.

HAECs were treated with axitinib, and ROS levels were measured by lucigenin‐enhanced chemiluminescence. Axitinib increased ROS production at 5 (*P* = 0.006; *n* = 6), 30 (*P* = 0.045; *n* = 6) and 60 min (*P* = 0.0002; *n* = 6) compared with vehicle‐treated cells (*n =* 6; Figure 1a). ROS levels were also elevated long term (24 h) following axitinib treatment (*P* = 0.0001; *n =* 6), whereas no significant differences observed at 4 (*P* = 0.283; *n =* 6) and 8 h (*P* = 0.692; *n =* 6; Figure 1b).

Given that oxidative stress can activate PARP, we next assessed PARP activity. PARP activity was significantly higher in axitinib‐treated cells compared with vehicle controls at 15 (*P* = 0.001; *n =* 7) and 30 min (*P* = 0.015; *n =* 7; Figure 1c, d, respectively). Co‐treatment with olaparib or tiron, a ROS scavenger [15 min: *P* = 0.030 and *P* = 0.025, respectively (*n =* 5); 30 min: *P* = 0.0009 (*n =* 7) and *P* = 0.004 (*n =* 6), respectively], prevented the increase in PARP activity at both time points.

### 3.2 Axitinib decreases SIRT1 activity and increases p53 acetylation in human endothelial cells

**FIGURE 2:**
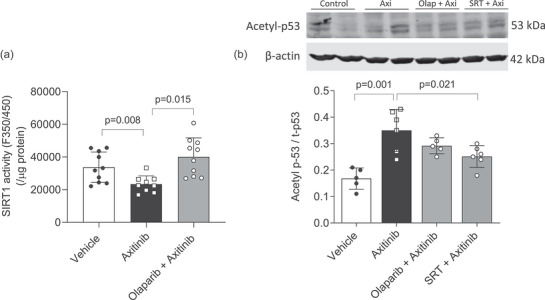
Axitinib decreases SIRT1 activity and increases p53 acetylation in human endothelial cells. (a) Human aortic endothelial cells were treated for 24 h with vehicle (*n =* 10), axitinib (*n =* 9) or axitinib plus olaparib (*n =* 10). SIRT1 activity was assessed by fluorometric assay. (b) Levels of acetylated p53 were evaluated by immunoblotting, and densitometric analysis was normalized to β‐actin (*n =* 5 or 6 different samples). Where indicated, cells were pretreated with olaparib or SRT1720 for 30 min before axitinib exposure [vehicle (*n =* 5); axitinib (*n =* 6); olaparib + axitinib (*n =* 5); SRT1720 + axitinib (*n =* 6)]. Results are expressed as the mean ± SD (one‐way ANOVA with *post hoc* test). SIRT1: sirtuin 1.

To determine whether VEGFi affects SIRT1 activity, HAECs were treated with axitinib. To investigate the role of PARP, some groups were also treated with the PARP inhibitor olaparib. Axitinib (*n =* 6) significantly reduced SIRT1 activity compared with vehicle‐treated cells (*P* = 0.008, *n =* 5; Figure 2a). The effect was no longer observed with co‐treatment with olaparib (*P* = 0.015, *n =* 10). Treatment with the SIRT1 activator SRT1720 was used as positive control and increased SIRT1 activity (data not shown).

Given that SIRT1 regulates p53 acetylation, we used levels of acetylated p53 as a downstream readout for SIRT1 activity. Consistent with reduced SIRT1 activity, axitinib treatment significantly increased (*P* = 0.001, *n =* 6) the acetylation of p53 compared with vehicle (*n =* 5). Co‐treatment with olaparib (*P* = 0.07, *n =* 5) or SRT1720 (*P* = 0.021, *n =* 6) reduced the axitinib‐induced increase in acetylated p53 (Figure 2b).

### 3.3 Effects of VEGFR inhibition on endothelial nitric oxide synthase activity

**FIGURE 3:**
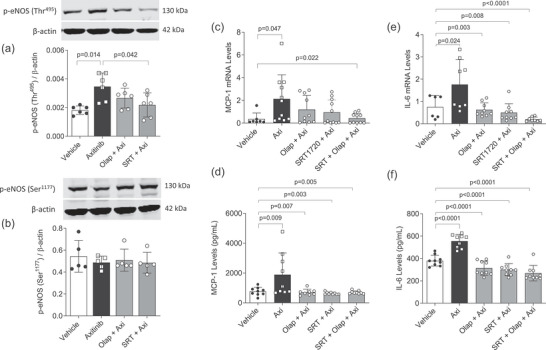
Axitinib reduces eNOS phosphorylation and increases pro‐inflammatory markers in human aortic endothelial cells (HAECs), which is reduced by PARP inhibition and SIRT1 activation. (a) Phosphorylation of eNOS at Thr^495^ was assessed by immunoblotting in HAECs, and densitometric analysis was normalized to β‐actin (*n =* 6 different samples). (b) Phosphorylation of eNOS at Ser^1^ ^1^ ^7^ ^7^ was found unchanged (*n =* 5). (c, e) *MCP‐1* and *IL‐6* mRNA levels were measured by RT‐qPCR in HAECs treated with vehicle (*n =* 7 and *n =* 6, respectively), axitinib (*n =* 11 and *n =* 8, respectively), axitinib plus olaparib (*n =* 10 and *n =* 9, respectively), axitinib plus SRT1720 (*n =* 11 and *n =* 9, respectively) or axitinib plus olaparib and SRT1720 (*n =* 11 and *n =* 8, respectively). (d, f) MCP‐1 and IL‐6 protein levels were assessed in cell culture medium by ELISA in the same treatment conditions (*n =* 9). Where indicated, olaparib and/or SRT1720 were added 30 min before axitinib exposure. Results are expressed as the mean ± SD (one‐way ANOVA with *post hoc* test). Olap: olaparib; Axi: axitinib; p‐eNOS: phosphorylated endothelial nitric oxide synthase; MCP‐1: monocyte chemoattractant protein‐1; IL‐6: interleukin‐6.

The molecular mechanisms underlying endothelial function were evaluated in HAECs by focusing on endothelial nitric oxide synthase (eNOS) activity, important in production of the vasodilator nitric oxide (NO). Axitinib significantly increased (*P* = 0.014) eNOS phosphorylation at Thr^495^ (inactive form of eNOS) in HAECs compared with vehicle‐treated cells (*n =* 6; Figure 3a). Co‐treatment with SRT1720 restored eNOS phosphorylation levels (*P* = 0.042, *n =* 6). Phosphorylation of eNOS at Ser^1^ ^1^ ^7^ ^7^ was found to be unchanged (*P* = 0.734, *n =* 5; Figure 3b).

### 3.4 Axitinib increases pro‐inflammatory markers in HAECs, which is reduced by PARP inhibition and SIRT1 activation

To investigate the functional impact of VEGFi on endothelial cells, we examined endothelial cell inflammatory responses, focusing on pro‐inflammatory signalling, MCP‐1 and IL‐6 expression. Axitinib (*n =* 11) increased *MCP‐1* mRNA levels (*P* = 0.047) compared with vehicle‐treated cells (*n =* 7; Figure 3c). Co‐treatment with olaparib, SRT1720, or their combination prevented the axitinib‐induced increase in *MCP‐1* expression (*n =* 10, *n =* 11, *n =* 11, respectively). Similar results were observed for *IL‐6* mRNA levels, with axitinib treatment inducing an increase (*P* = 0.024, *n =* 8) that was reduced by co‐treatment with olaparib, SRT1720, or both agents (*n =* 9, *n =* 9 and *n =* 8, respectively; Figure 3e).

Secreted levels of MCP‐1 and IL‐6 in the culture medium were also evaluated by ELISA. Axitinib increased MCP‐1 (*P* = 0.009) and IL‐6 (*P *< 0.0001) levels compared with vehicle. Co‐treatment with olaparib, SRT1720, or their combination prevented the axitinib‐induced increase in both MCP‐1 secretion (*P* = 0.003, *P* = 0.008 and *P *< 0.0001, respectively) and IL‐6 secretion (*P *< 0.0001 for all groups, *n =* 9 for all groups; Figure 3d, f).

### 3.5 PARP inhibition and SIRT1 activation reduce axitinib‐associated increase in adhesion molecules and THP‐1 adhesion to HAECs

**FIGURE 4:**
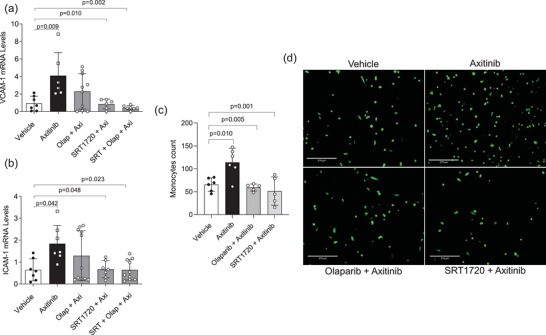
PARP inhibition and SIRT1 activation reduce axitinib‐associated increase in adhesion molecules and THP‐1 adhesion to human aortic endothelial cells (HAECs). (a, b) *VCAM‐1* and *ICAM‐1* mRNA levels were measured by RT‐qPCR in HAECs treated with vehicle (*n =* 7 for both), axitinib (*n =* 6 and *n =* 7, respectively), axitinib plus olaparib (*n =* 10 and *n =* 11, respectively), axitinib plus SRT1720 (*n =* 6 and *n =* 7, respectively) or axitinib plus olaparib and SRT1720 (*n =* 7 and *n =* 10, respectively). (c) Quantification of THP‐1 monocyte adhesion to HAECs following the same treatment conditions (*n =* 6 for vehicle and axitinib; *n =* 5 for olaparib + axitinib and SRT1720 + axitinib). (d) Representative fluorescence microscopy images of CFSE‐labelled THP‐1 monocytes adhering to HAEC are shown (×10 objective, green fluorescence). Where indicated, olaparib and/or SRT1720 were added 30 min before axitinib exposure. Results are expressed as the mean ± SD (one‐way ANOVA with *post hoc* test). Olap: olaparib; Axi: axitinib; VCAM‐1: vascular cell adhesion molecule‐1; ICAM‐1: intercellular adhesion molecule‐1.

To assess the effect of VEGFi on endothelial activation further, expression of adhesion molecules was measured in HAECs. Axitinib significantly increased *VCAM‐1* (*P* = 0.009) and *ICAM‐1* (*P* = 0.042) mRNA levels (*n =* 6 and *n =* 7, respectively) compared with vehicle‐treated cells (*n =* 7 for both). Co‐treatment with olaparib (*P* = 0.207, *n =* 10 and *P* = 0.574, *n =* 11, respectively), SRT1720 (*P* = 0.010, *n =* 6 and *P* = 0.049, *n =* 7, respectively), or their combination (*P* = 0.002, *n =* 7 and *P* = 0.023, *n =* 10, respectively) prevented axitinib‐induced upregulation of both *VCAM‐1* and *ICAM‐1* (Figure 4a, b).

We next evaluated monocyte adhesion to HAECs using CFSE‐labelled THP‐1 cells. Axitinib increased the number of adhered monocytes compared with vehicle‐treated cells (*n =* 6 for both groups; *P* = 0.010; Figure 4c). Co‐treatment with olaparib (*n =* 5, *P* = 0.005) or SRT1720 (*n =* 5, *P* = 0.01) significantly reduced THP‐1 adhesion in axitinib‐treated HAECs. Representative images of THP‐1 adhesion in each treatment condition are shown (Figure 4d).

### 3.6 Axitinib increases contractile responses in mesenteric arteries through mechanisms involving SIRT1 and PARP

**FIGURE 5:**
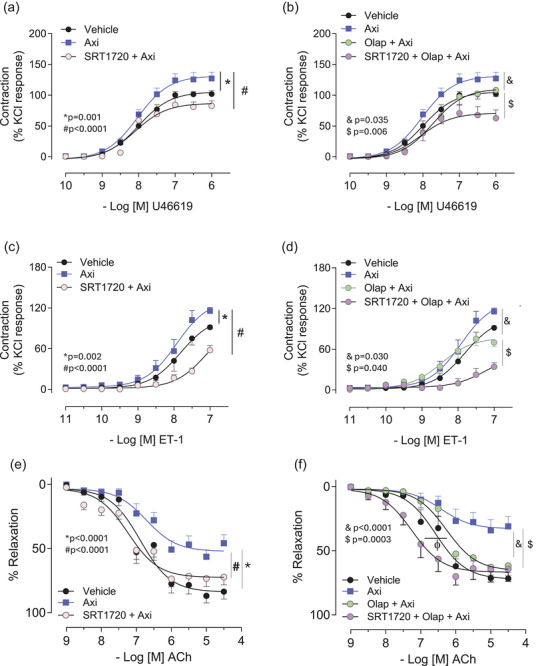
Axitinib increases contractile responses and impairs endothelium‐dependent relaxation in mesenteric arteries through a mechanism involving SIRT1 and PARP. (a, b) Concentration–response curves to the thromboxane analogue U46619 in mesenteric arteries isolated from C57BL/6 mice treated with vehicle (*n =* 10), axitinib (*n =* 11), axitinib plus SRT1720 (*n =* 8), axitinib plus olaparib (*n =* 4) or axitinib plus olaparib and SRT1720 (*n =* 8). (c, d) Concentration–response curves to endothelin‐1 (ET‐1) in the same treatment conditions [vehicle (*n =* 8), axitinib (*n =* 8), axitinib plus SRT1720 (*n =* 4), axitinib plus olaparib (*n =* 8) or axitinib plus olaparib and SRT1720 (*n =* 4)]. Contractions are expressed as a percentage of the response to 62.5 mM KCl. (e, f) Concentration–response curves to ACh in mesenteric arteries in similar conditions [vehicle (*n =* 10), axitinib (*n =* 10), axitinib plus SRT1720 (*n =* 8), axitinib plus olaparib (*n =* 7) or axitinib plus olaparib and SRT1720 (*n =* 7)]. Relaxation responses are expressed as a percentage of precontraction with U46619 (0.1 µM) (one vessel segment per mouse; non‐linear regression). Arteries were mounted in a wire myographand, where indicated, SRT1720 and/or olaparib was added 30 min before axitinib exposure. Results are expressed as the mean ± SD. ^*^ *P*‐values for comparing vehicle versus axitinib; ^#^ *P*‐values for comparing axitinib versus SRT1720 + axitinib; ^&^for comparing axitinib versus olaparib + axitinib; and ^$^for comparing axitinib versus axitinib SRT1720 + olaparib + axitinib. Olap: olaparib; Axi: axitinib; ACh: acetylcholine; ET‐1: endothelin‐1.

To assess the effects of VEGFi on vascular reactivity, isolated mouse mesenteric arteries were studied by myography. Axitinib enhanced contractile responses to the thromboxane analogue U46619 compared with vehicle‐treated arteries (Figure 5a). Co‐treatment with the SIRT1 activator SRT1720 attenuated the axitinib‐induced increase in contraction (*P *< 0.0001). Likewise, co‐treatment with olaparib and SRT1720, alone or in combination, prevented the enhanced contractile response to U46619 observed in axitinib‐treated arteries [vehicle (*n =* 10), axitinib (*n =* 11), axitinib plus SRT1720 (*n =* 8), axitinib plus olaparib (*n =* 4), or axitinib plus olaparib and SRT1720 (*n =* 8); Figure 5b].

To evaluate whether the contractile responses were generalized phenomena or specific to U46619, we studied effects of another vasoconstrictor, ET‐1. In a separate set of experiments, axitinib increased contractile responses to ET‐1 compared with vehicle (Figure 5c). Co‐treatment with SRT1720 attenuated the enhanced ET‐1 effects (*P *< 0.0001). Additionally, co‐treatment with olaparib, SRT1720, or their combination reduced ET‐1‐induced contractions in axitinib‐treated arteries [vehicle (*n =* 8), axitinib (*n =* 8), axitinib plus SRT1720 (*n =* 4), axitinib plus olaparib (*n =* 8), or axitinib plus olaparib and SRT1720 (*n =* 4); Figure 5d].

### 3.7 Axitinib impairs endothelium‐dependent relaxation

To evaluate the effects of VEGFi on endothelial function, mouse mesenteric arteries were mounted on a wire myograph, and responses to ACh were assessed. Axitinib treatment impaired ACh‐induced relaxation compared with vehicle‐treated arteries (*P *< 0.0001; Figure 5e). Co‐treatment with the SIRT1 activator SRT1720 improved relaxation responses (*P *< 0.0001). Likewise, co‐treatment with olaparib, alone or in combination with SRT1720, attenuated the axitinib‐induced endothelial dysfunction [vehicle (*n =* 10), axitinib (*n =* 10), axitinib plus SRT1720 (*n =* 8), axitinib plus olaparib (*n =* 7), or axitinib plus olaparib and SRT1720 (*n =* 7); Figure 5f].

## 4 DISCUSSION

Our findings provide new insights into molecular mechanisms underlying VEGFR inhibitor‐induced vascular injury. We identify redox‐sensitive processes involving activation of PARP and disruption of SIRT1 signalling as putative processes contributing to endothelial cell inflammation and vascular dysfunction. The rapid induction of oxidative stress observed in endothelial cells following axitinib treatment is in agreement with previous studies demonstrating that VEGF inhibitors promote redox imbalance in vascular tissues through upregulation of NADPH oxidases and inhibition of Nrf2‐regulated antioxidant systems, processes implicated in VEGF inhibitor‐induced vascular toxicity and hypertension (Neves et al., 2018; Pandey et al., 2018; Touyz et al., 2018). Here we evaluated responses to axitinib, but previous studies examined other VEGFR inhibitors, including sunitinib and sorafenib, which also induced vascular oxidative stress, indicating a VEGFi class effect rather than a drug‐specific effect (Faruque et al., 2014; le Noble et al., 2023; Lorenc et al., 2024; Luna et al., 2013). Additionally, we have previously reported that VEGFR inhibition is associated with PARP activation in vascular smooth muscle cells. In the present study, we advance this by: (1) providing evidence that PARP activation is redox sensitive; (2) identifying disruption of the SIRT1 axis as a mechanistic node downstream of PARP; (3) linking these molecular changes to eNOS inhibitory phosphorylation and to pro‐inflammatory markers; and (4) importantly, demonstrating translation to intact resistance arteries, where SIRT1 activation/PARP inhibition rescues vascular dysfunction.

The relationship between oxidative stress and PARP activation observed in our experiments provides mechanistic insight into how VEGFR inhibition triggers vascular dysfunction. PARP‐1, a DNA base repair enzyme, is activated by DNA breaks induced by oxidative and nitrosative stress (Alves‐Lopes et al., 2020, 2023; Wang et al., 2019). VEGFi‐induced production of ROS serves as a potent stimulus for PARP activation and DNA damage, as demonstrated by increased PARP activity at 15 and 30 min following axitinib exposure. This is consistent with previous findings that oxidative stress‐induced overactivation of PARP‐1 consumes substantial amounts of cellular NAD^+^ and, consequently, ATP, leading to cellular dysfunction and potential cell death (Wang et al., 2019). Our observation that olaparib prevented PARP activation in axitinib‐treated cells confirms involvement of this pathway.

The decrease in SIRT1 activity following axitinib treatment represents a crucial link in the pathophysiological cascade from VEGF signalling inhibition to vascular dysfunction. SIRT1 and PARP‐1 share NAD^+^ as an essential cofactor, creating competition for this limited metabolic resource (Luna et al., 2013). Upon activation, PARP‐1 catalyses the formation of poly(ADP‐ribose) polymers using NAD^+^ as a substrate, which depletes cellular NAD^+^ levels (Langelier et al., 2024; Luna et al., 2013). Given that SIRT1 requires NAD^+^ for its deacetylase activity, PARP activation directly competes with SIRT1 for available NAD^+^ (Bai, Cantó, Oudart et al., 2011). This NAD^+^ competition has been well documented, with studies showing that activation of PARP causes NAD^+^ depletion and inhibits SIRT1 activity (Bai, Cantó, Brunyánszki et al., 2011; Bai, Cantó, Oudart et al., 2011). The increased p53 acetylation we observed following axitinib treatment further supports the functional impairment of SIRT1, because SIRT1 deacetylates p53 (Solomon et al., 2006). The restoration of SIRT1 activity and reduction in p53 acetylation by olaparib treatment suggests that PARP inhibition might preserve NAD^+^ availability for SIRT1 function. However, the present study is limited by the lack of direct quantification of NAD^+^ levels. We also acknowledge the absence of direct SIRT1 activity measurements in the combined treatment groups as a limitation of the study, and future work will directly quantify SIRT1 activity and NAD^+^ availability to strengthen the mechanistic link between PARP activation and SIRT1 disruption. SIRT1 inhibitor (or genetic knockdown) approaches were not performed and will also be addressed in future work.

SIRT1 plays a fundamental role in regulating endothelial NO and endothelium‐dependent vascular tone by deacetylating eNOS (Mattagajasingh et al., 2007). Previous studies demonstrated that SIRT1 and eNOS colocalize and coprecipitate in endothelial cells and that SIRT1 deacetylates eNOS, stimulating its activity and increasing endothelial NO production (Bai et al., 2014; Mattagajasingh et al., 2007). Moreover, inhibition of SIRT1 in arterial endothelium has been shown to inhibit endothelium‐dependent vasodilatation and decrease bioavailable NO (Davis et al., 2013; Mattagajasingh et al., 2007). This supports our finding that axitinib reduced eNOS activity by increasing phosphorylation at Thr^495^, providing a molecular mechanism for the impaired vasodilatation observed in our *ex vivo* experiments. Although NO levels were not measured directly in the present study, future experiments directly assessing endothelial NO bioavailability will be important to substantiate the functional link between VEGFR inhibition, SIRT1 disruption and eNOS dysfunction. The impaired vasorelaxation and hypercontractile responses to U46619 and ET‐1 observed in axitinib‐treated vessels also demonstrate the functional consequences of this pathway. It is possible that these responses mimic the clinical presentation of hypertension in patients receiving VEGFi therapy. The improvement of these contractile responses by olaparib and SRT1720 indicates that PARP inhibition and SIRT1 activation can effectively counteract the injurious vascular effects of VEGF signalling inhibition.

VEGF normally exerts protective effects in the vasculature primarily through the production of vasodilatory mediators. When VEGF binds to VEGFR2, it triggers autophosphorylation and activates the PI3K/Akt pathway, leading to increased intracellular calcium and activation of eNOS (Olsson et al., 2006; Simons et al., 2016). The resultant increase in NO production promotes vascular permeability, endothelial cell survival and endothelium‐dependent vasodilatation (Roy et al., 2023). Our results showing increased ROS production and activation of redox‐sensitive pathways following axitinib administration suggested that downregulation of VEGFR signalling is associated with vasoinjurious processes. The prevention of ROS‐related effects by the antioxidant tiron in our study confirms the central role of oxidative stress in this pathophysiological cascade. The time‐dependent and non‐linear pattern of ROS accumulation following axitinib exposure might reflect an early oxidative burst induced by acute VEGFR blockade, followed by compensatory antioxidant responses and endothelial adaptation during sustained stress, consistent with established models of dynamic redox signalling in endothelial cells (Sies et al., 2017; Ungvari et al., 2018). Nonetheless, this aspect warrants further investigation.

The pro‐inflammatory state induced by axitinib, characterized by increased MCP‐1 and IL‐6 expression and secretion, represents another aspect of endothelial dysfunction resulting from VEGF signalling inhibition. This inflammatory response, along with the upregulation of adhesion molecules (VCAM‐1 and ICAM‐1) and increased monocyte adhesion, contributes to vascular pathology. Previous studies have demonstrated that PARP‐1 interacts with pro‐inflammatory transcription factors, including nuclear factor‐κB, increasing pro‐inflammatory mediators (Pazzaglia & Pioli, 2019; Vuong et al., 2015). The ability of both olaparib and SRT1720 to prevent these inflammatory changes suggests multiple protective mechanisms.

The clinical relevance of our findings is supported by observations that blood pressure is lower in patients treated with combination olaparib and bevacizumab in comparison to those receiving bevacizumab monotherapy, suggesting that PARP inhibition might attenuate VEGF inhibitor‐induced vascular dysfunction in clinical settings (Han et al., 2024). Given that cardiovascular toxicities are significant concerns with VEGF/VEGFR inhibitor therapy, with increased risk of hypertension, cardiac ischaemia, arterial thromboembolism and cardiac dysfunction, strategies to mitigate these effects while maintaining anticancer efficacy are crucial (Neves et al., 2018, 2023; Touyz et al., 2018). Our findings suggest several potential therapeutic approaches. Inhibiting oxidative stress by decreasing NADPH oxidase activity or activating Nrf2‐regulated antioxidant systems during VEGF inhibitor treatment might prevent cardiovascular risk and hypertension without compromising anticancer therapy. Additionally, PARP inhibition appears to be a promising approach to maintain NAD^+^ levels, preserve SIRT1 activity and protect against vascular dysfunction. This is supported by evidence from multiple disease models where PARP inhibition improved vascular function, prevented cardiomyocyte necrosis and reduced myocardial infarction size after cardiac reperfusion injury (Alves‐Lopes & Touyz, 2018; Alves‐Lopes et al., 2020; Qin et al., 2016; Song et al., 2008).

## 5 CONCLUSION

**FIGURE 6:**
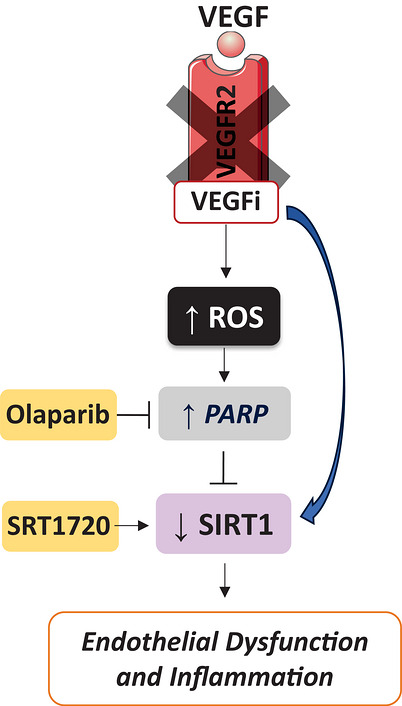
Schematic diagram showing possible mechanisms underlying endothelial dysfunction associated with VEGFR signalling inhibition and the role of the PARP–SIRT1 axis. We demonstrated that axitinib increases ROS production in human aortic endothelial cells, which is associated with activation of the PARP and disruption of SIRT1 signalling. Inhibition of PARP and activation of SIRT1 might represent an efficient approach to ameliorate vascular dysfunction observed in VEGFi‐treated cancer patients.

In conclusion, our results establish a mechanistic pathway connecting VEGFR signalling inhibition to vascular dysfunction through oxidative stress, PARP activation and disruption of SIRT1 signalling. VEGF/VEGFR inhibition increases oxidative stress, which activates PARP‐1. PARP‐1 reduces SIRT1 activity, which impairs eNOS function, ultimately leading to vascular dysfunction characterized by hypercontractility, impaired endothelium‐dependent relaxation and a pro‐inflammatory state (Figure 6). This pathway not only advances our understanding of the vascular toxicities of VEGFi therapy but also identifies potential targets for intervention to attenuate these effects while maintaining anticancer efficacy.

## AUTHOR CONTRIBUTIONS

All experiments were performed in the laboratory of Rhian M. Touyz. Karla B. Neves participated in the design, acquisition, analysis and interpretation of data for the work and in the drafting, review and editing the manuscript. Rheure Alves‐Lopes participated in the design, acquisition and analysis of data and in drafting of the work. Augusto C. Montezano contributed to the conceptualization, design, supervision and critical revision of the work. Rhian M. Touyz supervised and secured funding for the work and contributed to the design, review and editing of the work. All authors approved the final version of the manuscript and agree to be accountable for all aspects of the work in ensuring that questions related to the accuracy or integrity of any part of the work are appropriately investigated and resolved. All designated authors qualify for authorship, and all those who qualify for authorship are listed.

## CONFLICT OF INTEREST

None declared.

## Data Availability

All data supporting the results are included in the paper. Additional information about data or results from this study are available upon reasonable request.
